# Evaluating Cross-Resistance to Cry and Vip Toxins in Four Strains of *Helicoverpa armigera* With Different Genetic Mechanisms of Resistance to Bt Toxin Cry1Ac

**DOI:** 10.3389/fmicb.2021.670402

**Published:** 2021-05-14

**Authors:** Liangxuan Qi, Hanyang Dai, Zeng Jin, Huiwen Shen, Fang Guan, Yihua Yang, Bruce E. Tabashnik, Yidong Wu

**Affiliations:** ^1^Key Laboratory of Plant Immunity, College of Plant Protection, Nanjing Agricultural University, Nanjing, China; ^2^Department of Entomology, The University of Arizona, Tucson, AZ, United States

**Keywords:** cross-resistance, *Bacillus thuringiensis*, epistasis, resistance management, genetically engineered crops, cotton, cotton bollworm, complementation

## Abstract

Evolution of resistance by pests has diminished the efficacy of transgenic crops producing insecticidal proteins from *Bacillus thuringiensis* (Bt). In China, where transgenic cotton producing Bt toxin Cry1Ac has been planted since 1997, field control failures have not been reported but the frequency of resistance to Cry1Ac has increased in the cotton bollworm, *Helicoverpa armigera.* This provides incentive to switch to multi-toxin Bt cotton, which is grown in many other countries. Previous work created four laboratory strains of *H. armigera* with >100-fold resistance to Cry1Ac, with the genetic basis of resistance known in all but the LF256 strain. Here, we analyzed the genetic basis of resistance in Cry1Ac in LF256 and evaluated cross-resistance of all four strains to three toxins produced by widely planted multi-toxin Bt cotton: Cry1Fa, Cry2Ab, and Vip3Aa. DNA sequencing revealed that LF256 lacked the mutations in three genes (*HaTSPAN1*, *HaABCC2*, and *HaABCC3*) that confer resistance to Cry1Ac in two other strains of *H. armigera* we analyzed. Together with previous results, the data reported here show that each of the four strains examined has a different genetic basis of resistance to Cry1Ac. Significant positive cross-resistance occurred to Cry1Fa in three of the four strains tested but not to Cry2Ab or Vip3Aa in any strain. Thus, Cry2Ab and Vip3Aa are likely to be especially valuable for increasing the efficacy and durability of Bt cotton against *H. armigera* populations that have some resistance to Cry1Ac.

## Introduction

Environmentally friendly control of some key insect pests has been achieved with insecticidal proteins from the bacterium *Bacillus thuringiensis* (Bt) delivered via sprays for nearly a century and via genetically engineered crops since 1996 (Sanahuja et al., 2011). The hectares planted globally to transgenic Bt crops rose from 1 million in 1996 to over 108 million in 2019 (ISAAA, 2021). Bt crops can suppress pests and reduce the need for insecticide sprays, thereby providing economic and environmental benefits (National Academies of Sciences, Engineering, and Medicine [NASEM], 2016; Dively et al., 2018; Tabashnik et al., 2021). However, evolution of pest resistance to Bt crops has reduced such benefits (Tabashnik and Carrière, 2019). Some populations of at least nine major pests have evolved practical resistance to Bt crops, which is defined as field-evolved resistance that has practical consequences for pest control (Calles-Torrez et al., 2019; Smith et al., 2019; Tabashnik and Carrière, 2019).

Here, we focus on resistance to Bt toxins in the cotton bollworm, *Helicoverpa armigera*. This polyphagous lepidopteran is one of the world’s most devastating crop pests and has recently invaded the Americas (Kriticos et al., 2015; Anderson et al., 2018). In northern China, transgenic cotton producing Bt toxin Cry1Ac has been planted by millions of smallholder farmers since 1997 (Jin et al., 2018). Our previous study showed that in this region, the percentage of *H. armigera* larvae resistant to Cry1Ac has increased significantly, from 0.93% in 2010 to 5.5% in 2013 (Jin et al., 2015). A related study reported that the mean percentage of resistant individuals in China increased from 0% in 2006 and 2007 to 4.7% in 2017 (Zhang et al., 2018). These results provided evidence of an “early warning of resistance,” rather than practical resistance, because the percentage of resistant individuals was less than 50%, and reduced efficacy of Bt cotton in the field was not reported (Jin et al., 2015; Tabashnik and Carrière, 2017; Zhang et al., 2018).

While farmers in China continue to plant transgenic cotton producing only one Bt toxin (Cry1Ac), farmers in Australia, Brazil, India, the United States, and other countries have shifted to Bt cotton producing toxin combinations including Cry1Ac + Cry2Ab, Cry1Ac + Cry1F + Vip3Aa, and Cry1Ac + Cry2Ab + Vip3Aa (Sorgatto et al., 2015; Tabashnik and Carrière, 2017). A switch in China from single-toxin Bt cotton producing Cry1Ac to multi-toxin Bt cotton could help in delaying evolution of practical resistance to Bt cotton in *H. armigera* (Carrière et al., 2016). A better understanding of the mechanisms of resistance and patterns of cross-resistance between Cry1Ac and other toxins deployed in Bt cotton could be useful in making informed choices about the best toxins to use in such Bt cotton.

The most common mechanism of lepidopteran resistance to Cry toxins is disruption of their binding to larval midgut proteins (Peterson et al., 2017). High levels of resistance are conferred by mutations affecting cadherin, tetraspanin, and ABC transporter proteins (de Bortoli and Jurat-Fuentes, 2019; Heckel, 2020). Our previous work has documented >100-fold resistance to Cry1Ac in four laboratory strains of *H. armigera* from China: SCD-r1, SCD-KI, C2/3-KO, and LF256 (Table 1). We created SCD-r1 by introducing into susceptible strain SCD a naturally occurring recessive mutation that introduces a premature stop codon into a gene (*HaCad*) encoding a cadherin protein that binds Cry1Ac in the midgut of susceptible larvae (Xu et al., 2005; Yang et al., 2009). We produced SCD-KI by using CRISPR/Cas9 to knock into SCD a single-base pair substitution (T92C) in a gene (*HaTSPAN1*) encoding a tetraspanin protein (Jin et al., 2018). The T92C mutation causes non-recessive resistance to Cry1Ac in SCD-KI and occurs naturally in field populations of this pest in China (Jin et al., 2018; Guan et al., 2021). We created C2/C3-KO by using CRISPR/Cas9 to knock out two genes (*HaABCC2* and *HaABCC3*) in SCD encoding the ABC transporter proteins ABCC2 and ABCC3 (Wang J. et al., 2020). Although the specific mutations we introduced in these two genes have not been identified as occurring naturally, most naturally occurring *ABCC2* resistance alleles are knockouts caused by frameshifts or splicing aberrations (Heckel, 2020). The autosomal recessive resistance to Cry1Ac in LF256 was derived from a male moth (#256) captured in Langfang in northern China and incorporated in LF256 via a series of crosses, DNA screening, and selection with Cry1Ac (Gao et al., 2018). The genetic basis of resistance to Cry1Ac in LF256 is not known.

**TABLE 1:** Genetic basis of resistance to Cry1Ac in four strains of *H. armigera.*

**Strain**	**Gene(s) affected**	**Type of mutation**	**Reference(s)**
SCD-r1	*HaCad*	Premature stop codon	Xu et al., 2005; Yang et al., 2009
SCD-KI	*HaTSPAN1*	Substitution (T92C)	Jin et al., 2018
C2/3-KO	*HaABCC2* and *HaABCC3*	Deletion	Wang J. et al., 2020
LF256	Not known	Not known	Gao et al., 2018

In a previous interstrain complementation test for allelism, the first generation (F_1_) progeny from crossing LF256 and SCD-r1 were resistant to Cry1Ac, suggesting that the resistance in LF256 is associated with *HaCad*, as in SCD-r1 (Gao et al., 2018). However, further analysis refuted this hypothesis because resistance in LF256 was not genetically linked with *HaCad* and not associated with disruptive mutations in *HaCad*, changes in *HaCad* transcript abundance, or binding of Cry1Ac to cadherin (Gao et al., 2018). Overall, these results imply that epistasis between *HaCad* and one or more other loci conferring resistance in LF256 yielded the observed resistance in the F_1_ progeny from the complementation test (Gao et al., 2018). Previous work has not determined if the resistance in LF256 entails mutations affecting *HaTSPAN1*, *HaABCC2*, or *HaABCC3*.

Here, to better understand the genetic basis of resistance in LF256, we screened this strain for the T92C mutation in *HaTSPAN1* and for mutations in *HaABCC2* and *HaABCC3*. We also conducted complementation tests for allelism among the three strains with recessive resistance to Cry1Ac (SCD-r1, C2/C3-KO, and LF256). The results show that the genetic basis of resistance in LF256 differs from the other three strains, indicating that each of the four strains has a different mechanism of resistance. We also tested each of the four strains for cross-resistance to three toxins used in commercially available multi-toxin Bt cotton: Cry1Fa, Cry2Ab, and Vip3Aa. The results show significant positive cross-resistance to Cry1Fa in three of the four strains, but not to Cry2Ab or Vip3Aa in any strain.

## Materials and Methods

### Insect Strains and Rearing

The susceptible strain SCD was originally collected from Côte D’Ivoire (Ivory Coast, Africa) in the 1970s (Yang et al., 2009). It has been maintained in the laboratory without exposure to insecticides or Bt toxins for more than 40 years. The resistant strain SCD-r1 was established by introgression of the r1 allele of *HaCad* from the Cry1Ac-resistant GYBT strain into the SCD strain and has shown 440- to 540-fold resistance to Cry1Ac relative to SCD (Xu et al., 2005; Yang et al., 2009; Zhang et al., 2012). The resistant strain LF256 was isolated from the F_1_ offspring produced by a cross between field-captured male #256 from Langfang in the Hebei province of northern China and a female from SCD-r1 (Gao et al., 2018). The knockin strain SCD-KI was established by introducing the T92C mutation of *HaTSPAN1*, which was originally identified as a naturally occurring mutation, into the SCD strain via CRISPR/Cas9 (Jin et al., 2018). The knockout strain C2/C3-KO was created from the SCD strain by using CRISPR/Cas9 to knock out both *HaABCC2* and *HaABCC3* (Wang J. et al., 2020).

Larvae were reared on an artificial diet based on wheat germ and soybean powder (Shen and Wu, 1995) at 26 ± 1°C, 60 ± 10% relative humidity, and 16 h light and 8 h dark cycle. We supplied a 10% (w/v) sugar solution for adults.

### Bt Toxins

We bought Bt activated toxins Cry1Ac and Cry1Fa from Dr. Marianne Pusztai Carey (Case Western Reserve University, United States). The Institute of Plant Protection, Chinese Academy of Agricultural Sciences (CAAS), China, generously provided Cry2Ab and Vip3Aa protoxins.

### Bioassays

We used diet overlay bioassays to evaluate susceptibility of each strain to the four Bt toxins listed above. We diluted stock suspensions of each toxin with a 0.01 M, pH 7.4 phosphate buffer solution (PBS). Liquid artificial diet (1.2 ml) was dispensed into each well (surface area = 2 cm^2^) of a 24-well plate. After the diet cooled and solidified, 100 μl of Bt protein solution was applied evenly to the diet surface in each well. A single unfed neonate (24 h old) was put in each well after the Bt protein solution was dried at room temperature. After 7 days, larvae were recorded as survivors if they were alive and weighed >5 mg (Wang J. et al., 2020). We tested each of the five strains (four resistant strains and SCD) against four toxins: Cry1Ac, Cry1Fa, Cry2Ab, and Vip3Aa. In each of these 20 bioassays, we tested 48 larvae (24 × 2) at five to eight concentrations of each toxin (sample size for each bioassay = 240–384 larvae). In each of these 20 bioassays, as well as in the six bioassays for mode of inheritance and the six bioassays for complementation tests (see below), 48 larvae were put on untreated diet as controls, and the control mortality was consistently low (mean = 1.7%, range = 0–4.2%).

The concentration of Bt toxin killing 50% of larvae (LC_50_) and the 95% fiducial limits of the LC_50_ for each strain and toxin were calculated by probit analysis using PoloPlus (LeOra Software, 2002). Two LC_50_ values were considered significantly different based on the conservative criterion of no overlap between their 95% fiducial limits (Tabashnik et al., 1987; Payton et al., 2003).

### Sequencing of cDNA From *HaABCC2*, *HaABCC3*, and *HaTSPAN1*

To sequence the cDNA of *HaABCC2*, *HaABCC3*, and *HaTSPAN1*, the total RNA of the midgut tissue from fifth instars was individually extracted for each strain using the SV total RNA isolation system (Promega, Madison, WI, United States) according to the manufacturer’s instructions and reverse transcribed with the Moloney murine leukemia virus reverse transcriptase (Promega). Specific primers (Supplementary Table 1) and TaKaRa Premix Taq^TM^ (Shiga, Japan) were used to amplify three overlapping gene fragments of *HaABCC2* and *HaABCC3*, and a 187-bp cDNA fragment flanking the T92C position of *HaTSPAN1*. For *HaABCC2* and *HaABCC3*, PCR products of the expected size were cloned into the pGEM-T easy vector system (Promega) and sequenced by Tsingke (Beijing, China). We obtained full cDNA sequences of *HaABCC2* and *HaABCC3* from each of the 24 larvae, 12 from SCD and 12 from LF256. To screen for T92C of *HaTSPAN1*, PCR products from each of the 12 larvae from SCD and 12 from LF256 were purified and directly sequenced by Tsingke.

### *qRT-PCR* of cDNA From *HaABCC2* and *HaABCC3*

Total RNA isolation and synthesis of cDNA was performed as described above. Each sample used for qRT-PCR analysis was pooled from five midguts from fifth instars. We analyzed four samples from LF256 and four from SCD. Real-time PCR samples were prepared in SYBR^®^ Premix Ex Taq^TM^ (TaKaRa), and reactions were conducted with the primers shown in Supplementary Table 2 using the 7500 RT-PCR detection system (ABI, United States). qRT-PCR included an initial incubation of 30 s at 95°C followed by 40 cycles of amplification at 95°C for 5 s and 60°C for 34 s. We calculated transcript levels of *HaABCC2* and *HaABCC3* in LF256 relative to the SCD strain using the 2^–Δ^ ^Δ^ ^*CT*^ method (Livak and Schmittgen, 2001) with normalization based on the reference gene *EF-1α* (Yang et al., 2006).

### Mode of Inheritance

To evaluate dominance, maternal effects, and sex linkage, we used bioassays to determine responses to Cry1Ac of SCD-r1, C2/C3-KO, LF256, SCD, and the F_1_ progeny from reciprocal mass crosses between each of the three resistant strains and the susceptible strain SCD. For each of the six reciprocal crosses, we put 30 males of one strain and 30 females of the other strain in one cage to produce F_1_ progeny. Ninety-six unfed neonates (24 h old) of F_1_ progeny from each of the four strains and each of the six crosses between strains were tested in bioassays with the diagnostic concentration of Cry1Ac (0.1 μg per cm^2^ diet), which kills virtually all larvae from the susceptible SCD strain. We used percentage survival at this concentration to calculate the dominance parameter *h*, which varies from 0 to 1 for completely recessive to completely dominant resistance (Liu and Tabashnik, 1997): *h* = (survival of F_1_ progeny - survival of SCD)/(survival of resistant strain - survival of SCD). We did not include SCD-KI in the mode of inheritance tests or complementation tests (below) because its inheritance of resistance is not recessive (*h* = 0.56, Jin et al., 2018), which means that complementation tests with this strain would not be useful (Gao et al., 2018).

### Complementation Tests

We conducted complementation tests for allelism as described previously for *H. armigera* (Gao et al., 2018) for each of the three pairs of strains with recessive resistance to Cry1Ac (SCD-r1 and C2/3-KO, SCD-r1 and LF256, and C2/C3-KO and LF256). For each of the two reciprocal crosses between strains, 30 females of one strain and 30 males of the other strain were put in one cage to produce F_1_ progeny. From the F_1_ progeny from each of the six crosses, we tested 96 neonates on diet treated with the diagnostic concentration of Cry1Ac (0.1 μg per cm^2^ diet).

As described previously (Gao et al., 2018), if the recessive resistance alleles occur at one locus in one parent and a different locus in the other parent of a mating pair, their F_1_ progeny will be heterozygous for resistance at both loci. In this case, assuming no epistatic interactions between the two loci, the progeny are expected to be susceptible because of “allelic complementation” in which the dominant allele for susceptibility at each locus “complements” the recessive allele for resistance at each locus and restores the wild-type phenotype (i.e., susceptible). Conversely, if the recessive resistance alleles occur at the same locus in both parents, complementation does not occur. The progenies are resistant because they inherit two resistance alleles at the same locus.

To quantify the results of the complementation tests, we calculated the index of commonality (*C*), which measures the extent to which resistance alleles in two resistant strains are expected to share a common locus (Zhang et al., 2012). Values of *C* close to 0 indicate that the resistance alleles in the two strains do not share a common locus. Values close to 1 indicate that, in the absence of epistasis, the resistance alleles in the two strains are expected to share a common locus (Zhang et al., 2012; Gao et al., 2018). If the recessive alleles conferring resistance in each strain in the complementation test do not occur at a common locus and no epistasis occurs between the resistance-conferring loci in the two strains, zero survivors are expected. We used Fisher’s exact test to determine if the observed proportion of survivors differed significantly from zero.

## Results

### Magnitude of Resistance to Cry1Ac

Relative to the susceptible SCD strain, the resistance ratios for Cry1Ac were 540 for SCD-r1, 180 for SCD-KI, >5,160 for C2/3-KO, and 145 for LF256 (Table 2), consistent with previous results for these strains (Yang et al., 2009; Gao et al., 2018; Jin et al., 2018; Wang J. et al., 2020).

**TABLE 2:** Responses to Cry1Ac by larvae from the susceptible SCD strain and four resistant strains of *H. armigera*.

**Strain**	**RR^a^**	**LC_50_ (95% FL)^b^**	**Slope ± SE^c^**
SCD	1.0	0.0031 (0.0023–0.0040)	1.7 ± 0.2
SCD-r1	540	1.67 (1.30–2.23)^d^	1.4 ± 0.2
SCD-KI	180	0.56 (0.40–0.89)^d^	1.1 ± 0.2
C2/3-KO	>5,160^e^	>16^e^	NA^f^
LF256	145	0.45 (0.33–0.65)^d^	1.2 ± 0.2

*^*a*^Resistance ratio = LC_50_ of Cry1Ac for strain/LC_50_ of Cry1Ac for SCD.*

*^*b*^Concentration of Cry1Ac killing 50% and its 95% fiducial limits in μg toxin per cm^2^ diet, *n* = 288 to 336 larvae for each LC_50_.*

*^*c*^Slope of the concentration – mortality line and its standard error.*

*^*d*^Significant difference between the LC_50_ for a strain versus SCD based on the conservative criterion of no overlap of the 95% fiducial limits.*

*^*e*^No mortality (*n* = 48) at the highest concentration tested: 16 μg Cry1Ac per cm^2^ diet.*

*^*f*^Not available.*

### Strain LF256: *HaTSPAN1*, *HaABCC2*, and *HaABCC3*

**FIGURE 1:**
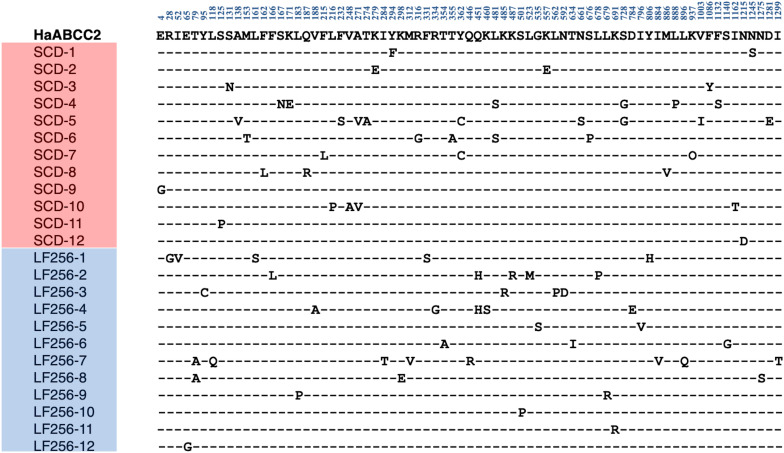
Alignment of polymorphic amino acids predicted from sequencing cDNA of *HaABCC2* from 12 larvae of susceptible strain SCD and 12 larvae of Cry1Ac-resistant strain LF256 of *H. armigera.* Dashes indicate the amino acids are the same as in the SCD GenBank sequence (MW592372, top line in bold).

**FIGURE 2:**
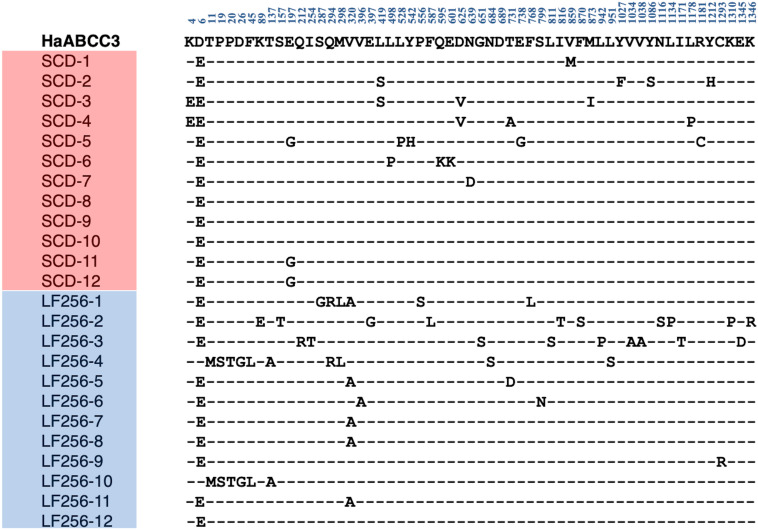
Alignment of polymorphic amino acids predicted from sequencing cDNA of *HaABCC3* from 12 larvae of susceptible strain SCD and 12 larvae of Cry1Ac-resistant strain LF256 of *H. armigera.* Dashes indicate the amino acids are the same as in the SCD GenBank sequence (MW592373, top line in bold).

Direct sequencing of *HaTSPAN1* PCR products from 24 larvae, 12 from LF256 and 12 from SCD, revealed that all 24 lacked the T92C mutation that confers non-recessive resistance to Cry1Ac in SCD-KI. Sequencing of full-length cDNA of *HaABCC2* and *HaABCC3* (Figures 1, 2) from another 24 larvae, 12 from SCD and 12 from LF256, revealed no predicted amino acid insertions, deletions, or premature stop codons in LF256 relative to SCD. Although the predicted amino acid sequence varied within LF256 and SCD for both genes, we found no consistent differences between strains in the predicted sequence for either *HaABCC2* or *HaABCC3* (Figures 1, 2). Analysis of transcript abundance by qRT-PCR revealed no significant difference between LF256 and SCD for either *HaABCC2* (*t-*test, *t* = 1.55, df = 3, *P* = 0.24) or *HaABCC3* (*t*-test, df = 3, *t* = 0.67, *P* = 0.68). Relative to SCD with a standardized mean of 1.0, the mean transcript abundance was not lower for LF256 for either *HaABCC2* (1.4, SE = 0.2) or *HaABCC3* (1.2, SE = 0.2). Thus, the results imply that the genetic basis of resistance in LF256 differs from that of SCD-KI and C2/3-KO.

### Mode of Inheritance

**FIGURE 3:**
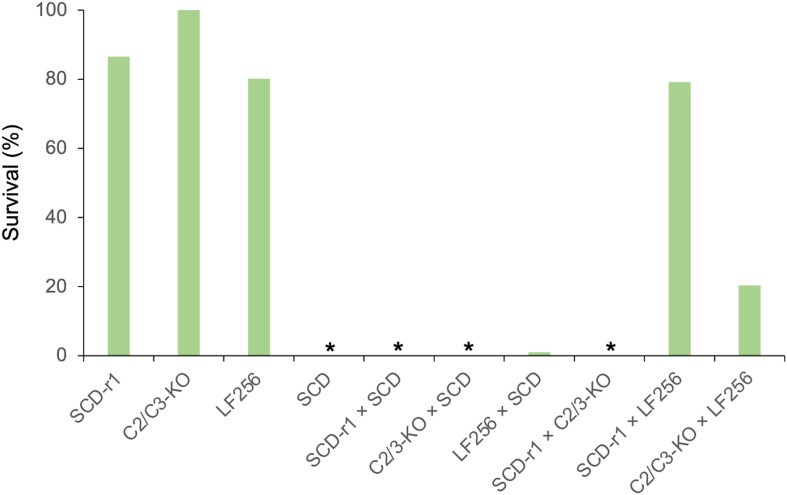
Survival at the diagnostic concentration of Cry1Ac of *H. armigera* larvae from three resistant strains (SCD-r1, C2/C3-KO, and LF256), a susceptible strain (SCD), and the F_1_ progeny from crosses between strains. Asterisks indicate 0% survival for SCD and the progeny from three crosses: SCD-r1 × SCD, C2/3-KO × SCD, and SCD-r1 × C2/3-KO. Survival for progeny from LF256 × SCD was 1.0%.

The results here show that resistance to Cry1Ac was autosomal and recessive in SCD-r1, C2/3-KO, and LF256 (Figure 3), which confirms previous conclusions (Yang et al., 2009; Gao et al., 2018; Wang J. et al., 2020). For each of these three strains, we detected no differences in responses between the progeny from reciprocal crosses with SCD (Supplementary Table 3), indicating that maternal effects and sex linkage were not evident. For these strains, inheritance of resistance at 0.1 μg of Cry1Ac per cm^2^ diet was recessive with *h* = 0 for SCD-r1 and C2/3-KO and *h* = 0.01 for LF256 (Figure 3 and Supplementary Table 3).

### Complementation Tests Between Strains With Recessive Resistance to Cry1Ac

In the complementation test between SCD-r1 and C2/3-KO, larval survival on diet with 0.1 μg of Cry1Ac per cm^2^ diet was 0% for the F_1_ progeny, with the index of commonality (*C*) = 0 (Figure 3 and Supplementary Table 3). This result implies that these two strains do not share a locus at which mutations confer resistance, consistent with the molecular evidence showing that resistance is conferred by a mutation in *HaCad* for SCD-r1 and knockout of *HaABCC2* and *HaABCC3* in C2/3-KO (Yang et al., 2009; Wang J. et al., 2020).

By contrast, the complementation test results show that larval survival on treated diet for F_1_ progeny was 79.2% for the cross between LF256 and SCD-r1 and 20.3% for the cross between LF256 and C2/3-KO, with *C* = 0.94 and 0.22, respectively (Figure 3 and Supplementary Table 3). In both cases, the observed proportion of survivors is greater than zero (Fisher’s exact test, *n* = 192 and *P* < 0.0001 for each complementation test). These results imply that LF256 shares a common resistance locus with SCD-r1 and C2/3-KO, epistasis occurred between the resistance loci in LF256 and each of the other two strains, or both.

### Cross-Resistance to Cry1Fa, Cry2Ab, and Vip3Aa

Cross-resistance to Cry1Fa was >100-fold in C2/3-KO, 3.1-fold in SCD-r1, and 2.5-fold in SCD-KI (Table 3). The cross-resistance to Cry1Fa was statistically significant in each of these three strains based on the conservative criterion of non-overlap of the 95% fiducial limits of the LC_50_ values between each resistant strain and SCD (Table 3). However, LF256 was not cross-resistant to Cry1Fa (resistance ratio = 0.9, Table 3).

**TABLE 3:** Responses to Cry1Fa, Cry2Ab, and Vip3Aa by larvae from the susceptible SCD strain and four Cry1Ac-resistant strains of *H. armigera*.

**Strain**	**Bt toxin**	**RR^a^**	**LC_50_ (95% FL)^b^**	**Slope ± SE^c^**
SCD	Cry1Fa	1.0	0.49 (0.40–0.59)	2.4 ± 0.3
SCD-r1	Cry1Fa	3.1	1.51 (0.97–2.64)^d^	1.5 ± 0.2
SCD-KI	Cry1Fa	2.5	1.21 (0.93–1.63)^d^	1.5 ± 0.2
C2/3-KO	Cry1Fa	>100^e^	>50^e^	NA^f^
LF256	Cry1Fa	0.9	0.43 (0.29–0.59)	2.0 ± 0.2
SCD	Cry2Ab	1.0	0.14 (0.09–0.19)	2.0 ± 0.2
SCD-r1	Cry2Ab	1.4	0.20 (0.15–0.25)	1.6 ± 0.2
SCD-KI	Cry2Ab	1.2	0.17 (0.14–0.21)	2.0 ± 0.2
C2/3-KO	Cry2Ab	0.2	0.028 (0.022–0.033)^d^	3.1 ± 0.4
LF256	Cry2Ab	1.4	0.19 (0.15–0.23)	2.1 ± 0.2
SCD	Vip3Aa	1.0	0.20 (0.17–0.25)	2.3 ± 0.2
SCD-r1	Vip3Aa	1.5	0.29 (0.22–0.36)	2.5 ± 0.3
SCD-KI	Vip3Aa	1.0	0.20 (0.15–0.26)	1.9 ± 0.2
C2/3-KO	Vip3Aa	1.3	0.25 (0.12–0.50)	2.9 ± 0.3
LF256	Vip3Aa	0.9	0.18 (0.14–0.22)	2.1 ± 0.3

*^*a*^Resistance ratio = LC_50_ for strain/LC_50_ for SCD for the same toxin.*

*^*b*^Concentration killing 50% and its 95% fiducial limits in μg toxin per cm^2^ diet, *n* = 240 to 384 larvae for each LC_50_.*

*^*c*^Slope of the concentration – mortality line and its standard error.*

*^*d*^Significant difference between the LC_50_ for a strain versus SCD based on the conservative criterion of no overlap of the 95% fiducial limits.*

*^*e*^No mortality (*n* = 24) at the highest concentration tested: 50 μg Cry1Fa per cm^2^ diet.*

*^*f*^Not available.*

In SCD-r1, SCD-KI, and LF256, cross-resistance to Cry2Ab was not significant (resistance ratio = 1.2–1.4, mean = 1.3, Table 3). By contrast, C2/C3-KO had fivefold, statistically significant negative cross-resistance to Cry2Ab (Table 3).

No significant difference in the LC_50_ of Vip3Aa occurred between SCD and each of the four Cry1Ac-resistant strains (resistance ratio = 0.9–1.5, mean = 1.2, Table 3).

## Discussion

The new results reported here include cDNA sequences of *HaABCC2*, *HaABCC3*, and *HaTSPAN1* from resistant strain LF256 of *H. armigera*, comparison of transcript abundance between LF256 and susceptible strain SCD for *HaABCC2* and *HaABCC3*, complementation tests between LF256 and resistant strain C2/3-KO, and evaluation of cross-resistance to Cry1Fa, Cry2Ab, and Vip3Aa in four resistant strains of *H. armigera* (zero, three, and one of the four resistant strains were previously tested for cross-resistance to these toxins, respectively). Together with previous results, the new results demonstrate that each of the four Cry1Ac-resistant strains of *H. armigera* we analyzed (LF256, SCD-KI, C2/3-KO, and SCD-r1) has a different genetic basis of resistance. The results here show that the 145-fold resistance to Cry1Ac of LF256 was not associated with the T92C mutation in *HaTSPAN1* that causes non-recessive resistance to Cry1Ac in strain SCD-KI (Jin et al., 2018). Also, transcript abundance for *HaABCC2* and *HaABCC3* did not differ significantly between LF256 and susceptible strain SCD. Moreover, the results here show that relative to SCD, LF256 did not have insertions, deletions, premature stop codons, or consistent differences in predicted amino acid sequences encoded by *HaABCC2* or *HaABCC3.* Thus, resistance to Cry1Ac in LF256 is not associated with mutations disrupting *HaABCC2* or *HaABCC3*, unlike strain C2/C3-KO where resistance to Cry1Ac is caused by CRISPR-mediated knockout of both of these genes (Wang J. et al., 2020). Previous results indicate that LF256 lacks disruptive mutations in *HaCad* and is not genetically linked with *HaCad* (Gao et al., 2018). This differs from strain SCD-r1, where a premature stop codon in *HaCad* confers resistance to Cry1Ac (Xu et al., 2005; Yang et al., 2009). Although the specific genetic basis of resistance to Cry1Ac in LF256 remains to be identified, we conclude it differs from strains SCD-KI, C2/3-KO, and SCD-r1.

In contrast with the molecular evidence summarized above, the complementation results show that survival was significantly greater than 0% in the F_1_ progeny of crosses between LF256 and C2/3-KO (20.3%) and between LF256 and SCD-r1 (79.2%). The index of commonality (*C*) for LF256 and SCD-r1 was 0.94 here, similar to 0.91 reported previously (Gao et al., 2018). Considered alone, the complementation results would suggest a high probability of a shared resistance locus between LF256 and SCD-r1 and a moderate probability of a shared resistance locus between LF256 and C2/3-KO. However, the molecular data demonstrate that SCD-r1, C2/3-KO, and LF256 do not share a common locus at which mutations confer resistance to Cry1Ac. Thus, we hypothesize that epistasis caused the observed survival in the F_1_ progeny of the crosses between LF256 and C2/3-KO here and between LF256 and SCD-r1 here and previously (Gao et al., 2018).

The observed results could reflect epistasis mediated by interactions between various midgut proteins involved in the mode of action of Bt toxins (Gao et al., 2018). Although the genetic basis of resistance in LF256 could involve two or more loci, we use the term unidentified LF256 resistance locus for simplicity. The complementation results reported here and previously imply that a high proportion of individuals heterozygous for resistance at both *HaCad* and the unidentified LF256 resistance locus are resistant (Gao et al., 2018). Thus, the unidentified locus acts like a modifier by effectively increasing the dominance of resistance associated with the mutation in *HaCad*. This premature stop codon confers completely recessive resistance when it occurs in a susceptible genetic background, as seen in the cross between SCD-r1 and SCD. Because resistance evolves faster as dominance increases (Tabashnik et al., 2004; Tabashnik and Carrière, 2017), the epistasis between *HaCad* and the unidentified locus is expected to accelerate evolution of resistance (Gao et al., 2018).

Resistance to Cry1Fa seen here was nil in LF256, 2.5-fold in SCD-KI, 3.1-fold in SCD-r1, and >100-fold in C2/3-KO, which had both *HaABCC2* and *HaABCC3* knocked out. Similarly, in our work with *Plutella xylostella*, knocking out both *PxABCC2* and *PxABCC3* caused >10,000-fold resistance to Cry1Ac and 380-fold resistance to Cry1Fa (Zhao et al., 2021). However, knocking out either *PxABCC2* or *PxABCC3* alone caused at most 2.9-fold resistance to Cry1Ac and no resistance to Cry1Fa (Zhao et al., 2021). By contrast, in *Spodoptera exigua*, knocking out *SeABCC2* alone caused >470-fold resistance to Cry1Ac and 240-fold resistance to Cry1Fa, whereas knocking out *SeABCC3* alone did not cause resistance to either toxin (Huang et al., 2020). For the congeneric species *Spodoptera frugiperda*, knocking out *SfABCC2* yielded resistance ratios of 182 for Cry1Ab and 124 for Cry1Fa versus 16.5 for Cry1Ab and 34.5 for Cry1Fa caused by knocking out *SfABCC3* (Jin et al., 2020). In *Ostrinia furnacalis*, knocking out *OfABCC2* caused eightfold resistance to Cry1Ac and >300-fold resistance to Cry1Fa (Wang X. et al., 2020). Thus, the roles of *ABCC2* and *ABCC3* in mediating toxicity of Cry1Ac or Cry1Ab and Cry1Fa vary among the five species of Lepidoptera mentioned above.

The statistically significant fivefold increase in susceptibility to Cry2Ab in C2/3-KO is unexpected and the only one of the 12 cases examined here where the Cry1Ac-resistant strain was significantly more susceptible than SCD to any of the other toxins tested. Negative cross-resistance between Bt toxins is rare, and weak positive cross-resistance usually occurs between Cry1Ac and Cry2Ab (Carrière et al., 2015; Welch et al., 2015; Tabashnik and Carrière, 2019). More specifically, excluding C2/3-KO, the mean resistance to Cry2Ab in Cry1Ac-resistant strains of *H. armigera* was 1.3-fold in this study (1.2-fold in SCD-KI and 1.4-fold in both SCD-r1 and LF256) and 2.7-fold (range: 1.1- to 5.9-fold) in previous studies of five other strains of this pest selected in the lab for resistance to Cry1Ac (Supplementary Table 2 of Carrière et al., 2015). We hypothesize that the increased susceptibility to Cry2Ab is associated specifically with knockout of *HaABCC2* and *HaABCC3* in C2/3-KO, which definitely did not occur in SCD-KI, SCD-r1, or LF256, and probably not in the five other strains tested previously. The mechanism underlying increased susceptibility to Cry2Ab in C2/3-KO is not known. It might entail increased abundance of a receptor for Cry2Ab (e.g., *HaABCA2*, Wang et al., 2017) compensating for the lack of HaABCC2 and HaABCC3, elimination of binding of Cry2Ab to HaABCC2 and HaABCC3 that reduces toxicity of Cry2Ab, or both. It remains to be determined if naturally occurring mutations in *HaABCC2* and *HaABCC3* that confer resistance to Cry1Ac cause negative cross-resistance to Cry2Ab.

We found statistically significant positive cross-resistance to Cry1Fa in three of the four strains tested but not to Cry2Ab or Vip3Aa in any strain. This greater cross-resistance between Cry1Ac and Cry1Fa than between Cry1Ac and the other two toxins is consistent with previously reported results for many species (Carrière et al., 2015; Chakroun et al., 2016; Wei et al., 2019; Tabashnik and Carrière, 2020). This finding supports the hypothesis that cross-resistance is associated with amino acid sequence similarity between toxins (Tabashnik et al., 1996; Carrière et al., 2015) because amino acid sequence similarity is higher between Cry1Ac and Cry1Fa (60% overall) than between Cry1Ac and Cry2Ab or Cry1Ac and Vip3Aa (Supplementary Table 4; Carrière et al., 2015). However, cross-resistance was similarly low to Cry2Ab and Vip3Aa, despite the 37% overall amino acid sequence similarity between Cry1Ac and Cry2Ab versus no structural homology between Cry1Ac and Vip3Aa (Supplementary Table 4; Carrière et al., 2015).

Among the three toxins we evaluated for cross-resistance, the results here confirm that Cry2Ab and Vip3Aa are likely to be the most durable for deployment in Bt cotton to manage *H. armigera* populations that have some resistance to Cry1Ac. However, the results suggest that Cry1Fa could also be useful because only one of the four mechanisms of resistance studied here (knockout of both *HaABCC2* and *HaABCC3*) was associated with strong cross-resistance to this toxin. Moreover, the non-recessive T92C mutation in *HaTSPAN1* is expected to spread faster than recessive resistance mutations (Jin et al., 2018), but it conferred only a 2.5-fold resistance to Cry1Fa in SCD-KI. In principle, Bt cotton producing Vip3Aa, Cry2Ab, and Cry1Fa could be deployed in China to achieve more sustainable pest management.

## Data Availability Statement

The datasets presented in this study can be found in online repositories. The names of the repository/repositories and accession number(s) can be found below: https://www.ncbi.nlm.nih.gov/, MW592372, MW592373.

## Author Contributions

LQ, HD, YY, and YW conceived and designed the experiments. LQ, HD, ZJ, HS, and FG conducted the experiments. LQ, HD, YY, YW, and BT analyzed the data and wrote the manuscript. All authors read and approved the manuscript.

## Conflict of Interest

BT is co-author of a patent on modified Bt toxins, “Suppression of Resistance in Insects to *Bacillus thuringiensis* Cry Toxins, Using Toxins that do not Require the Cadherin Receptor.” BASF, Bayer CropScience, and Corteva did not provide funding to support this work, but may be affected financially by publication of this article and have funded other work by BT. The remaining authors declare that the research was conducted in the absence of any commercial or financial relationships that could be construed as a potential conflict of interest.
