# Supplementary material for: Evaluating Cross-Resistance to Cry and Vip Toxins in Four Strains of Helicoverpa armigera With Different Genetic Mechanisms of Resistance to Bt Toxin Cry1Ac
Source: Front Microbiol. 2021 May 14;12:670402. doi: 10.3389/fmicb.2021.670402 (PMC8160511; doi:10.3389/fmicb.2021.670402)
Supplement: Supplementary file 1 [file Data_Sheet_1.docx]

Supplementary Material

**TABLE S1**

Primers used to obtain full-length cDNA of *HaABCC2* and *HaABCC3* and to screen cDNA for the T92C mutation of *HaTSPAN1.*

**TABLE S2**

Primers used to analyse transcript levels of *HaABCC2* and *HaABCC3* by qRT-PCR.

**TABLE S3**

Survival at the diagnostic concentration of Cry1Ac of *H. armigera* larvae from a susceptible strain (SCD), three resistant strains (SCD-r1, C2/C3-KO, and LF256), and the F_1_ progeny from crosses between strains.

**TABLE S4**

Amino acid sequence similarity for pairs of Bt toxins used in pyramids.

**TABLE S1**. Primers used to obtain full-length cDNA of *HaABCC2* and *HaABCC3* and to screen cDNA for the T92C mutation of *HaTSPAN1.*

| Fragment name | Sense primer sequence (5'-3') | Reverse primer sequence (5'-3') | Use |
| --- | --- | --- | --- |
| *ABCC2-1* | ATTCGCGTGTCGCCCTTCAC | CATTTCAAAGGCACGAGCTG | Obtain full-length cDNA of *HaABCC2* |
| *ABCC2-2* | CGCGTGTCGCCCTTCTGGCT | CCTGTATTTGAGCCTTCTGT |  |
| *ABCC2-3* | TTCGCGTGTCGCCCTTACCA | TTCGCGTGTCGCCCTTGCCT |  |
| *ABCC3-1* | GCGTGTCGCCCTTCGCGGAA | GCAGCGGGTAGAAGACATTG | Obtain full-length cDNA of *HaABCC3* |
| *ABCC3-2* | TTCGCGTGTCGCCCTTCAAT | GCGTGTCGCCCTTCTCCTCG |  |
| *ABCC3-3* | TCGAATTCGCGTGTCGCCCT | ATATCGAATTCGCGTGTCGC |  |
| *HaTSPAN1* | AGTGGTGAACAATGCGGGAA | CTGCAGAACCGGACTCGTAG | Screen for T92C mutation of *HaTSPAN1* |

| **TABLE S2**. Primers used to analyse transcript levels of *HaABCC2* and *HaABCC3* by qRT-PCR.   \| Target gene \| Forward primer (5’-3’) \| Reverse primer (5’-3’) \| \| --- \| --- \| --- \| \| *HaABCC2* \| gtccagtcctggtgtgtctggt \| ccaatataactccgccgaaa \| \| *HaABCC3* \| GACAGAAGCGTGCAGATGAG \| GCAGCGGGTAGAAGACATTG \| |
| --- | --- | --- | --- | --- | --- | --- | --- | --- | --- |

**TABLE S3**. Survival at the diagnostic concentration of Cry1Ac of *H. armigera* larvae from a susceptible strain (SCD), three resistant strains (SCD-r1, C2/C3-KO, and LF256), and the F_1_ progeny from crosses between strains.

| Strain or cross | Survival % ^a^ | *h ^b^* | *C* ^c^ |  |
| --- | --- | --- | --- | --- |
| SCD | 0.0 |  |  |  |
| SCD-r1 | 86.5 |  |  |  |
| C2/C3-KO | 100.0 |  |  |  |
| LF256 | 80.2 |  |  |  |
|  |  |  |  |  |
| *Mode of inheritance tests* |  |  |  |  |
| SCD-r1♂ × SCD♀ | 0.0 |  |  |  |
| SCD-r1♀ × SCD♂ | 0.0 |  |  |  |
| Mean | 0.0 | 0.00 |  |  |
|  |  |  |  |  |
| C2/3-KO♂ × SCD♀ | 0.0 |  |  |  |
| C2/3-KO♀ × SCD♂ | 0.0 |  |  |  |
| Mean | 0.0 | 0.00 |  |  |
|  |  |  |  |  |
| LF256♂ × SCD♀ | 1.0 |  |  |  |
| LF256♀ × SCD♂ | 1.0 |  |  |  |
| Mean | 1.0 | 0.01 |  |  |
|  |  |  |  |  |
| *Complementation tests* |  |  |  |  |
| SCD-r1♀ × C2/3-KO♂ | 0.0 |  |  |  |
| SCD-r1♂ × C2/3-KO♀ | 0.0 |  |  |  |
| Mean | 0.0 |  | 0.0 |  |
|  |  |  |  |  |
| SCD-r1♀ × LF256♂ | 80.2 |  |  |  |
| SCD-r1♂ × LF256♀ | 78.1 |  |  |  |
| Mean | 79.2 |  | 0.94 |  |
|  |  |  |  |  |
| C2/3-KO♀ × LF256♂ | 20.8 |  |  |  |
| C2/3-KO♂ × LF256♀ | 19.8 |  |  |  |
| Mean | 20.3 |  | 0.22 |  |
| ^a^ Larvae were tested on diet treated with 0.1 μg Cry1Ac per cm^2^ diet. Sample size for means is 192 larvae, n = 96 for all other values. | | | | |
| ^b^ Dominance of resistance, varies from 0 for completely recessive resistance to 1 for completely dominant resistance. | | | | |
| ^c^ Index of commonality between resistant strains, see Materials and Methods. | | | | |
|  |  |  |  |  |

| **TABLE S4**. Amino acid sequence similarity for pairs of Bt toxins used in pyramids. | | | | | | |
| --- | --- | --- | --- | --- | --- | --- |
| Toxin pair | |  | Amino acid sequence similarity (%) ^a, b^ | | |  |
|  |  |  | Domain I | Domain II | Domain III | Overall |
| Cry1Ac | Cry1Fa | | 75 | 51 | 50 | 60 |
| Cry1Ac | Cry2Ab | | 42 | 15 | 40 | 37 |
| Cry1Fa | Cry2Ab | | 43 | 22 | 31 | 35 |

^a^ Vip3Aa has no structural homology with Cry toxins.

^b^ Source: Carrière et al. (2015).
